# Efficiency of Artificial Insemination at Natural Estrus in Organic *Churra* Ewes

**DOI:** 10.3390/vetsci9070370

**Published:** 2022-07-18

**Authors:** Carlos Palacios, José A. Abecia, Javier Plaza, Cristina Hidalgo, Luis F. de la Fuente

**Affiliations:** 1 Faculty of Environmental and Agricultural Sciences, University of Salamanca, Avenida Filiberto Villalobos 119-129, 37007 Salamanca, Spain; pmjavier@usal.es; 2 Institute of Research in Environmental Sciences of Aragón (IUCA), University of Zaragoza, Miguel Servet 177, 50013 Zaragoza, Spain; alf@unizar.es; 3 Faculty of Economic and Business Science, University of León, Campus de Vegazana s/n, 24071 León, Spain; cristina.hidalgo@unileon.es; 4 Faculty of Veterinary Medicine, University of León, Campus de Vegazana s/n, 24071 León, Spain; f.fuente@unileon.es

**Keywords:** artificial insemination, sheep, natural estrus, fertility, organic farming

## Abstract

**Simple Summary:**

Hormonal treatments used to synchronize estrus in sheep artificial insemination procedures can cause several physiological problems that can affect negatively fertility and animal health; however, alternative protocols based on the detection of natural estrus offer a more sustainable option while reaching successful fertility results. Therefore, in this study, an artificial insemination protocol at natural estrus in organic *Churra* sheep was performed. In the protocol design phase, 125 ewes were exocervically inseminated, and their fertility was assessed based on the number of inseminations, physiological state, body condition, estrus detection–insemination interval and vaginal fluids in cervix. That protocol was repeated in six consecutive years. In all ewes, fertilities based on the timing of insemination after estrus detection were very high. Lactating ewes produced better results than did dry ewes, which was probably because of the better feeding of the former. In addition, double insemination increased the fertility of ewes, whose estrus was detected within 16 h of onset. Body condition and amount of vaginal fluid were correlated with fertility. In conclusion, exocervical inseminations at natural estrus can produce acceptable fertility and prolificity in *Churra* ewes, avoiding physiological problems derived from the use of hormonal treatments.

**Abstract:**

Hormonal treatments used in the artificial insemination (AI) of sheep can cause several physiological problems that can affect negatively fertility and animal health; however, AI protocols based on the detection of natural estrus offer a more sustainable option and can achieve high fertility. In this study, an AI protocol at natural estrus in organic *Churra* sheep was performed. In the first phase (AI protocol development), 125 ewes were exocervically inseminated, and their fertility was assessed based on the following factors: number of AI, physiological state, body condition, estrus detection–AI interval, and vaginal fluids in cervix. That protocol was repeated for six consecutive years. In all individuals, fertilities based on the timing of insemination after estrus detection were very high. Lactating ewes produced better results than did dry ewes, which was probably because of the better feeding of the former. In addition, double insemination increased the fertility of ewes whose estrus was detected within 16 h of onset. Body condition and amount of vaginal fluid were correlated with fertility. Exocervical inseminations at natural estrus can produce acceptable fertility and prolificity in Churra ewes.

## 1. Introduction

In Spain, artificial insemination (AI) in sheep is used as an improvement genetic method within purebred selection programs. Livestock farms that are in the herd book of a breed can benefit from the selection scheme of that breed. To do so, they use AI to test males of high genetic value [1], rebreed their offspring, and confirm production by yield control. In Spain, the approved protocol for AI in sheep is the synchronization of estrus through the insertion of a vaginal sponge that has been impregnated with progestogens and, after 14 d, the removal of the sponge and the administration of intramuscular injectable pregnant mare’s serum gonadotropin (PMSG), which is currently known as equine chorionic gonadotropin (eCG). Insemination is performed 56 h after removal of the sponges if performed vaginally and 58–72 h if performed laparoscopically [2]. Although that procedure facilitates the simultaneous insemination of a large number of ewes, hormonal treatments can cause several physiological problems [3] such as the production of antibodies against eCG [4], alteration of vaginal flora, and changes in follicular dynamics [5]. Furthermore, the exocervical placement method does not increase fertility very much because there are difficulties in introducing semen intrauterinally, which is primarily because of the morphology of the ovine cervix [6]. Furthermore, it requires high coordination with the semen collection center because the semen should be refrigerated at 15 °C, and changes in temperature are factors that affect negatively the efficacy of the method. In addition, the number of doses available and the quality of the semen of the males that may have been used in the genetic center on that day are important factors [7].

The current regulations on organic farming [8] prohibit the use of hormones for animal growth stimulation and for the synchronization of estrus, although AI is allowed; however, this process is considered unfeasible in organic sheep and goats because hormones cannot be used to synchronize females. Sheep farms that are in selection schemes that intend to transform their management system to organic production are forced to abandon conventional AI procedures, even if they want to remain in the herd book. Thus, farmers need to adopt an AI process that does not involve hormonal treatments and detects the natural estrus of ewes over several days to identify the individuals susceptible to insemination. In some cases, AI is performed at natural estrus that uses light treatments to synchronize the estrus of ewes [9,10]. In addition, in the sexual season or estrus, the so-called male or ram effect can synchronize estrus [3,11,12]. In general, AI at natural estrus is more successful if conducted in the reproductive season rather than in the anestrus period [13]. Unlike cows, however, ewes do not exhibit signs that reflect the state of estrus; therefore, a means of detection is essential [13].

The main objective of this study was to quantify the efficiency of AI performed at natural estrus in *Churra* sheep under the European Organic certification. We hypothesized that AI procedures based on sheep natural estrus can produce high fertility without increasing economic costs, which would eliminate the need for other procedures that require the administration of hormones for estrus synchronization.

## 2. Materials and Methods

The research was performed in an organic *Churra* sheep farm in northwestern Spain that was part of the *Churra* breed selection scheme. The farm was surrounded by the *Los Arribes* del Duero Natural Park, near Portugal (41°24′23″ N–6°15′38″ W). The dehesa ecosystem predominated the area, with its typical semi-arid Mediterranean climate and poor-quality siliceous soils, which make the area ideal for extensive livestock farming. Specifically, the sheep flock was reared under a semi-extensive management system because the ewes spent nights outdoors, although weaned animals were milked twice a day for at least three months after lambing.

The first two years of the 8-year experiment were used to develop the best AI protocol, and in the remaining years, the established AI protocol was repeated. The Ethic Committee for Animal Experiments of the University of Salamanca approved the procedures performed in this study. Furthermore, the care and use of animals were in accordance with the Spanish Policy for Animal Protection (RD 53/2013), which meets the European union Directive 2010/63 on the protection of animal used for experimental and other scientific purposes.

### 2.1. AI Protocol Design

An AI program must be established well in advance of estrus detection because of the availability and time needed to collect semen as well as its limited shelf life. In this study, the process occurred at the “Ovigen” Genetic Improvement and Selection Center (Zamora, Spain), 60 km from the organic farm. Semen from adult Churra rams with proven fertility was collected daily by means of an artificial vagina, and selection of the rams was coordinated by the National Association of Sheep Breeders of the Churra Breed (ANCHE). The testing procedures of the extracted semen included pH, color, mass activity (motility score assessed on a 0–5 scale in which 0 = nulled motion and 5 = rapid motion), and concentration (spermatozoa per ml of semen). Following Moss et al. [14], only ejaculates that had a mass activity > 3 and a concentration > 3 × 10^9^ spermatozoa/mL were accepted for insemination. After that test, following the method of Evans and Maxwell [15], fresh semen was diluted with Tris-fructose egg yolk (2% egg yolk, *v*/*v*) diluent at a concentration of 8 × 10^8^ spermatozoa/mL, cooled until 15 °C, and loaded into mini-plastic straws (0.25 mL, 2 × 10^8^ spermatozoa/dose).

In general, an AI protocol for natural estrus involves several phases; specifically, selection of dates for insemination, method of detection of natural estrus in ewes, optimal time from the detection of estrus to perform insemination, and expected fertility. In Spain, the optimal reproductive season for sheep is in September and October, and the entire experiment was conducted in these months.

To detect estrus in the ewes in a flock of 700 sheep, three vasectomized rams with harness markers with chalk for marking mounted ewes were used. Following Gibbons and Cueto [16], rams were vasectomized by the removal of 2 cm of the vas deferens. AI was performed at 1300 h. Three groups of ewes were identified based on the moment the ewes were marked by a vasectomized ram: G1 ewes were marked between 21:00 h and 08:00 h the following day (5–16 h between estrus detection and insemination), G2 ewes were marked between 08:00 h and 13:00 h (<5 h between estrus detection and insemination), and G3 ewes were marked between 13:00 h and 21:00 h (16–24 h between estrus detection and insemination). To identify the ewes in each group, the color of the chalk on the vasectomized rams was changed each time a group of ewes was identified. Thereafter, 125 ewes were exocervically inseminated: 55 in the first year and 70 in the second year, and all between 11 and 24 September (14 d). Semen plastic straws were brought to the farm on each of the 14 days. In addition, among the 55 ewes inseminated in the first year, 36 were subjected to a second insemination 24 h after the first, which assessed the efficiency of this procedure as a means of increasing fertility. Thus, ewes in G1, G2, and G3 were re-inseminated 29–40 h, <29 h, and 40–48 h post estrus detection, respectively. Immediately before insemination, ewes were assessed for body condition on a scale 1 to 5 [17], the presence of vaginal fluid (absent, a few, medium, or much), and the productive group to which each ewe belonged, which was related to its physiological state, distinguishing among lactating (less than three months after lambing) or “dry” (not-lactating ewes that were not pregnant (more than three months after lambing).

Following Glowatzki-Mullis [18], the Animal Production Department of the Faculty of Veterinary Medicine of the University of León (Spain) performed the paternity analyses based on 18 microsatellite markers. To avoid lambing assignment errors, 15 d after insemination, the lambings of the ewes were recorded by electronic animal identification readers. To confirm paternity, the offspring born from the lambings of the inseminated ewes were subjected to a paternity test.

### 2.2. AI Experiment

Given the complexity and operational limitations of having to perform inseminations for each of the three groups of ewes based on when they were marked by the vasectomized males (G1, G2 and G3), the AI process of groups G2 and G3 were combined into a single group (G23), and therefore, they contained ewes that were inseminated between 16 and 29 h after the detection of estrus. Once the AI protocol was accepted, it was implemented in the farm in six consecutive years. In that period, 581 ewes ($n_{1}=70;\;n_{2}=205;\;n_{3}=45;\;n_{4}=66;\;n_{5}=87;\;n_{6}=108$) were exocervically inseminated on 47 d between September and October. The semen used in the experiment came from 62 rams that had been selected by the Ovigen Genetic Improvement and Selection Center (Zamora, Spain).

### 2.3. Statistical Analyses

The results of the first two years in which the AI protocol was developed were assessed based on fertility and prolificity. Given the high variation in fertility, it was necessary to assess the multinomial association that each of the factors—i.e., number of inseminations (single or double), physiological state (lactating or dry), body condition (1–5), number of hours post estrus detection until insemination (G1, G2, or G3), and vaginal fluid in the cervix (absent, a few, medium or much)—had on fertility. To that end, and given that all of the previous factors were categorical variables, a SAS Proc CATMOD (categorical models) [19], originally designed by Grizzle et al. [20], was performed. In addition, a Chi-square test ($\chi^{2}$) was used to assess the statistical significance of the relationship between the results and each of the factors, which was performed with SAS/STAT software v14.3 (SAS Institute Inc., Madrid, Spain). The results of the last six years in which the designed AI protocol was performed were expressed as fertility and prolificity efficiency. To assess the effect of the factors on fertility, particularly, male genetics and estrus detection—AI interval, a Chi-square test ($\chi^{2}$) was performed using IBM-SPSS Statistics v.26 package software (IBM, Chicago, IL, USA).

## 3. Results and Discussion

### 3.1. AI Protocol Design Procedure

Fertility (lambings/inseminated ewes) and prolificity (lambs/ewe) of the first two years in which the AI protocol was developed are shown in Table 1. In the first two years of the study in which the AI protocol was developed, average fertility was 0.24 and 0.40, respectively, (Table 1), which was similar to those reported in other *Churra* populations and other Spanish breeds that have the same type of semen but received induced estrus, e.g., 0.31 in the *Churra* breed [21] and 0.39 in the Assaf breed [7] but less than the 0.46 found in the *Castellana* breed [22] and the 0.45 in the *Rasa Aragonesa* breed [23]. Mean fertilities in the two years were much lower than those obtained from Lacaune ewes that received induced estrus and refrigerated semen (0.67) [24]. In addition, fertilities were similar to those of natural estrus inseminations that range from about 0.40 in meat breeds in South America [13,25].

Daily variability in fertility was high in the first year (from 0 to 0.38); however, except for the first day of the year of the study, daily fertilities were similar, although the daily sample size was small. The null result on the first day might indicate a failure with the marking method, because the semen and the application technique were the same on all days. Mean prolificacy rates were 1.23 and 1.25 in the first and second year, respectively, which were acceptable for ewes that did not receive hormonal stimulation.

Multinomial associations between the factors and fertility indicated by the CATMOD suggest that none of the factors included in the analysis had a significant association with fertility over the rest of the factors (Table 2); therefore, it is concluded that all the factors are equally associated with fertility.

The mean fertility of ewes that were inseminated either once (0.38) or twice (0.42) did not differ significantly (*p* > 0.05) (Table 3), which is similar to the results of Muñoz et al. [26].

The effect of the second insemination was most evident in ewes in the G1 group, which showed a 0.08 increase in fertility. In the ewes in the G3 group (inseminated later after estrus detection), fertility in the first and second insemination did not differ significantly. It would be interesting to perform double inseminations on ewes that have been detected in estrus no later than 16 h after onset. Furthermore, our results suggest that an early first insemination (<16 h) is less effective than a late insemination (16–24 h) [27].

Lactating ewes had a higher fertility than did dry ewes, but the difference was not statistically significant (Table 4). Others have suggested that non-lactating ewes have higher fertility because they have a longer inter-lambing interval than do lactating ewes [28]; however, others [29] have suggested that the key to high fertility is in the feeding because ewes that have an optimal energy intake, which are normally those in lactation, have higher fertility than do those that do not receive an energy supplement. In our study, the dry ewes were pasture-fed only.

The reproductive performance of ewes subjected to AI is regulated largely by nutritional state, which can be evaluated visually based on body condition. In our study, all of the ewes had body scores between 2 and 4 on the 5-point scale (Table 5). The results were similar to those of Carvalho et al. [30], who indicated that the extremes on the body condition scale are correlated with worse fertility.

Fertility in each of the three groups defined based on the number of hours elapsed since estrus detection until insemination did not differ significantly (Table 6), although late first inseminations produced higher fertility outcomes than did early inseminations [27]. Similar results were found in Corriedale ewes [26].

Analysis of vaginal fluid in the cervix area is essential for a successful AI procedure because the quantity and physicochemical characteristics dictate whether spermatozoa can pass through the cervix and fertilize the ovum [31].

Although fertility and vaginal fluid volume were not correlated significantly, the highest fertility occurred among the ewes that had an average volume of vaginal fluid, and the lowest fertility was among the ewes that had very high fluid volumes (Table 7). Other studies have obtained similar results and, besides being statistically significant [31], they have identified that the highest fertilities are obtained in ewes that have intermediate or small amounts of vaginal fluids in the cervix.

### 3.2. AI Protocol Experiment

**Figure 1:**
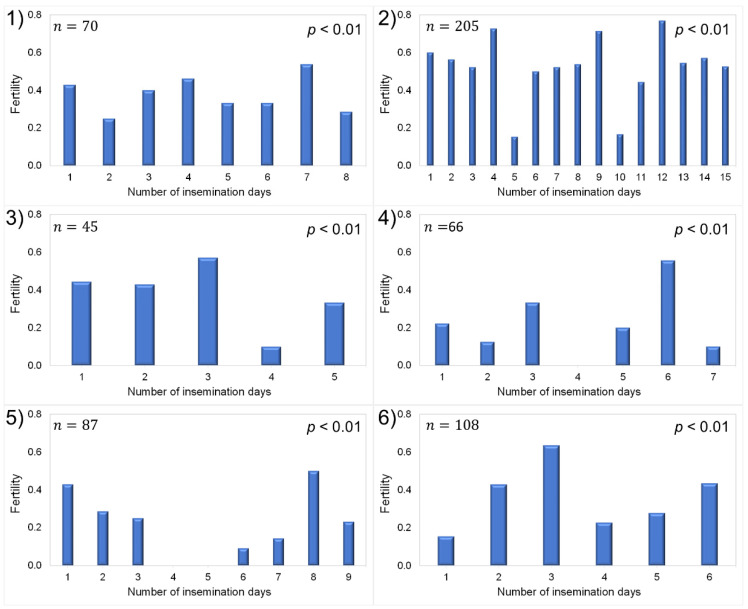
Fertility results of organic *Churra* ewes in Spain in six years of insemination (**1**–**6**) for each of the insemination days; *n*: number of ewes inseminated. Fertility values range between 0 and 1.

The average fertility in the last six years of the study was 0.39 (Figure 1), which is similar to the results of Salamon et al. [13] and Buckrell [25] for natural estrus inseminations, and it is within the range of 0.38–0.45 that has been reported in the *Churra* breed for induced estrus AI protocols [31]. In addition, fertility was more regular within years than it was between years, which has been reported elsewhere [27].

**Figure 2:**
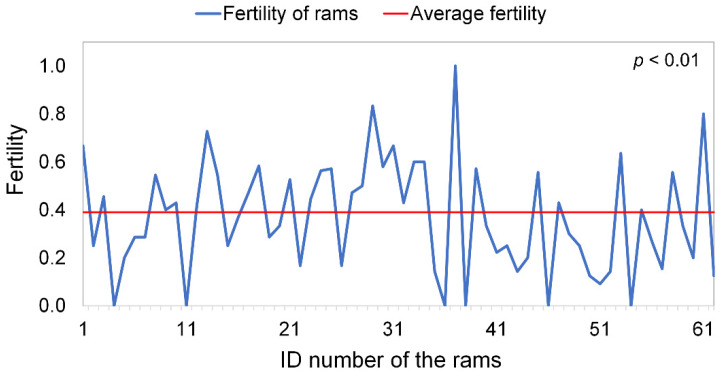
Fertility produced from the semen from each of 62 *Churra* rams in Spain.

The number of ewes inseminated by the AI protocol developed in our study per ram was very small because it depended on the number of ewes marked on that day and on the availability of semen from the individual ram (Figure 2). The number of ewes inseminated per ram ranged from 1 to 26. This, together with the inherent variability of the rams, meant that the resulting fertility differed significantly (*p* < 0.01) among rams. About a quarter (24.19%) of the rams had fertilities < 0.20, 25.80% had fertilities between 0.20 and 0.40, 45.16% had fertilities between 0.40 and 0.80, and 4.8% of the rams had fertilities > 80%. Male fertility is influenced by a multitude of intrinsic factors such as animal age and semen factors characteristics [21,32]; however, the factors related to ewes are as much or more important to fertility. The origin of the animals, the weather season in which they are inseminated [33], the protein nutritional inputs received by the ewes during the insemination process [34], and even weather conditions on the day of insemination [35] are other external factors that influence fertility. All of those factors, together with the small number of ewes inseminated per ram, explain the extreme variations in fertility per ram and insemination day.

In the six years in which the AI protocol was implemented, overall fertility was very stable, and the fertility of ewes marked by vasectomized rams at night (G1) (0.38) and those marked in the day (G23) (0.39) did not differ significantly (*p* = 0.451). Average fertilities among the last six years of the study differed significantly (*p* < 0.01) (Table 8), and the highest occurred in year 2 and the lowest occurred in year 5. In one year, only, fertility differed significantly (*p* < 0.05) between ewes that differed in the duration of the estrus detection-AI interval (Table 8).

Differences in fertility among the six years were similar to the results of Kukovics et al. [36] in Awassi, Merino, and Lacaune sheep. Furthermore, given the many factors that influence fertility, the scientific literature revealed fertility values associated with AI protocols from 0.18 [37] to intermediate values of 0.42 [38] and even reaching very high values of 0.65–0.75 [15].

Regarding the economic costs associated with AI at natural estrus, they are generally reduced to the daily travel of the inseminator from the semen production and collection center to the farm for at least one week. However, this cost is offset by savings in the cost of hormonal treatments. Palacios 2010 [39] estimated that within a 70 km perimeter between the farm and the semen production and collection center, the AI protocol at natural estrus is more cost-effective than protocols that require the use of hormones for estrus synchronization.

## 4. Conclusions

The results of this study suggest that natural estrus insemination can substitute for induced estrus insemination without a reduction in fertility or an increase in economic cost. The only difference is an increase in the labor needed on the days of insemination. Fertilities were very high in all groups and did not differ significantly based on the timing of insemination after estrus detection. Lactating ewes produced better results than did dry ewes, which was probably because of the better feeding of the former. Double insemination increased the fertility of ewes whose estrus was detected within 16 h of onset. The other factors evaluated such as body condition and abundance of vaginal fluid in the cervix were correlated with fertility in ways that were similar to other studies, although differences were not statistically significant in our study.

The implementation of the AI protocol used in this study for six years confirmed that exocervical inseminations at natural estrus can provide acceptable fertility and prolificity.

## Figures and Tables

**Table 1:** Fertility and prolificity of inseminations in organic *Churra* ewes in the first two years of a 8-year study on a farm in Spain.

Year	AI Date	Inseminated Ewes	Lambing	Lambs	Fertility	Prolificity (Lambs/Ewe)
					(0–1)	
1	17-Sep.	10	0	0	0.00	0.00
	18-Sep.	10	2	3	0.20	1.50
	19-Sep.	7	2	2	0.29	1.00
	20-Sep.	8	3	3	0.38	1.00
	21-Sep.	8	3	4	0.38	1.33
	24-Sep.	12	3	4	0.25	1.33
	Total	55	13	16	0.24	1.23
2	10-Sep.	7	3	3	0.42	1.00
	11-Sep.	4	1	1	0.25	1.00
	12-Sep.	5	2	2	0.40	1.00
	15-Sep.	13	6	9	0.46	1.50
	16-Sep.	9	3	4	0.33	1.33
	17-Sep.	12	4	6	0.33	1.50
	18-Sep.	13	7	10	0.53	1.42
	19-Sep.	7	2	2	0.28	1.00
	Total	70	28	35	0.40	1.25

AI: artificial insemination.

**Table 2:** CATMOD results for correlations between factors and fertility in organic *Churra* ewes in Spain.

Parameter	CATMOD Statistical Value	Significance *p*-Values
Number of AI	0.14	0.43
Physiological state	3.01	0.08
Body condition	3.19	0.20
Estrus detection–AI interval	0.14	0.93
Vaginal fluid in cervix	2.70	0.44

AI: artificial insemination.

**Table 3:** Number of AI procedures and fertility in organic *Churra* ewes in Spain.

Number of AI	Estrus Detection–AI Interval	Inseminated Ewes	Lambing	Lambs	Fertility	Significance
					(0–1)	*p*-Value
1st AI	G1 (16–5 h)	23	8	11	0.34	0.621
	G2 (<5 h)	11	5	6	0.37	
	G3	21	9	12	0.42	
	(24–16 h)					
	Total	55	22	28	0.38	
2nd AI	G1	14	6	7	0.43	
	(40–29 h)					
	G2 (<29 h)	10	4	6	0.40	
	G3	12	5	7	0.42	
	(48–40h)					
	Total	36	15	20	0.48	

**Table 4:** Physiological state and fertility of organic *Churra* ewes in Spain.

Physiological State	Inseminated Ewes	Lambing	Fertility (0–1)	*p*-Value
Lactating	59	28	0.48	0.103
Dry	66	19	0.29	

**Table 5:** Body condition and fertility in organic *Churra* ewes in Spain.

Body Condition Score	Inseminated Ewes	Lambing	Fertility (0–1)	*p*-Value
2	53	14	0.26	0.197
3	63	30	0.48	
4	9	4	0.40	

**Table 6:** Estrus detection—AI interval (h) and fertility in *Churra* ewes in Spain.

Estrus Detection—AI Interval	Inseminated Ewes	Lambing	Lambs	Fertility (0–1)	*p*-Value
G1	21	8	18	0.38	0.992
G2	70	25	30	0.37	
G3	34	14	19	0.41	

G1, G2 and G3: 5–16 h, <5 h, and 16–24 h between estrus detection and insemination, respectively. AI: artificial insemination.

**Table 7:** Volume of vaginal fluids in the cervix and the fertility of organic *Churra* ewes in Spain.

Vaginal Fluid Volume	Inseminated Ewes	Lambing	Lambs	Fertility (0–1)
Absent	10	4	5	0.37
Few	40	16	22	0.40
Medium	45	19	26	0.43
Much	30	5	7	0.16

**Table 8:** Estrus detection—AI interval and fertility in organic *Churra* ewes in the last six years of an 8-year study on a farm in Spain.

Year	Estrus Detection–AI Interval	Fertility (0–1)	*p*-Value
1	G1	0.38	0.441
	G23	0.42	
2	G1	0.54	0.499
	G23	0.53	
3	G1	0.30	0.233
	G23	0.46	
4	G1	0.19	0.404
	G23	0.28	
5	G1	0.18	0.517
	G23	0.23	
6	G1	0.46	0.029
	G23	0.26	

G1, G23: 16-5 h and 29-16 h between estrus detection and insemination, respectively. AI: artificial insemination.

## Data Availability

The data in this study are available upon request from the corresponding author. The study did not include humans.
